# Revealing Localised Mechanochemistry of Biomaterials Using *In Situ* Multiscale Chemical Analysis

**DOI:** 10.3390/ma15103462

**Published:** 2022-05-11

**Authors:** Nicholas T.H. Farr

**Affiliations:** 1 Department of Materials Science and Engineering, Sir Robert Hadfield Building, Mappin Street, University of Sheffield, Sheffield S1 3JD, UK; n.t.farr@sheffield.ac.uk; 2 Insigneo Institute for In Silico Medicine, The Pam Liversidge Building, Sir Robert Hadfeld Building, Mappin Street, Sheffield S1 3JD, UK

**Keywords:** mechanochemistry, biomaterials, advanced characterisation, surface analysis, scanning electron microscope, secondary electron hyperspectral imaging, multiscale analysis, polymer chemistry, biotechnology, hyperspectral imaging

## Abstract

The study of mechanical and chemical phenomena arising within a material that is being subjected to external stress is termed mechanochemistry (MC). Recent advances in MC have revealed the prospect not only to enable a greener route to chemical transformations but also to offer previously unobtainable opportunities in the production and screening of biomaterials. To date, the field of MC has been constrained by the inability of current characterisation techniques to provide essential localised multiscale chemically mapping information. A potential method to overcome this is secondary electron hyperspectral imaging (SEHI). SEHI is a multiscale material characterisation technique applied within a scanning electron microscope (SEM). Based on the collection of secondary electron (SE) emission spectra at low primary beam energies, SEHI is applicable to the chemical assessment of uncoated polymer surfaces. Here, we demonstrate that SEHI can provide in situ MC information using poly(glycerol sebacate)-methacrylate (PGS-M) as an example biomaterial of interest. This study brings the use of a bespoke in situ SEM holder together with the application of SEHI to provide, for the first time, enhanced biomaterial mechanochemical characterisation.

## 1. Introduction

Materials are rarely deployed in static environments, rather they are exposed to diverse and varying stresses within dynamic environments. The study of mechanical and chemical phenomena arising within a material that is being subjected to external stress is termed mechanochemistry (MC). Applied examples of MC in materials science are numerous and include mechanical fracturing, chemical alterations of mechanically stressed materials, stress corrosion/cracking or enhanced oxidation, tribology and polymer degradation. Recent advances in MC have revealed the prospect of not only enabling a greener route to chemical transformations but also to offer previously unobtainable opportunities in the production and screening of materials [1,2].

A promising area of research associated with MC, which has the potential to realise significant benefits, is the development of future biomaterials [1,3,4]. In common with other material systems, the impact of MC interactions is dependent on the context of a material’s deployment [4,5]. It is expected that for many material systems applications, MC interactions will occur but result in limited effects that are not detrimental to a material’s function. However, for other applications, the specific deployment scenarios of biomaterials may result in MC interactions, particularly on the surface of the material, that give rise to major complications [4,6,7]. Studies have revealed that even nanoscale surface alternations can affect the suitability of a biomaterial implant due to the fact of biomaterial cell interactions occurring at the nano–micron surface scale [8]. Recent studies have previously shown the value of understanding the MC mechanisms involved in order to provide insights into the progression of implanted surgical mesh failures [4]. This multidisciplinary approach has highlighted the potential to provide not only answers as to why materials have failed in the past but also how MC can inform the development of future biomaterials. An analysis technique that can identify MC interactions at a localised scale and also measure these effects in situ is confidently expected to bring new understandings in biomaterial/cell interaction and lay the foundations for improved pre-deployment verification.

To date, the field of MC has been constrained by the inability of current characterisation techniques to provide essential localised multiscale chemically mapping information. A 2021 *Nature Review* [9] concluded that the unifying feature of mechanochemical phenomena may be the coupling between inertial motion at the microscale to the macroscale and changes in the chemical bonding, which can be realised by the dynamic coupling of multiple-length scales. The requirement for multiscale localised analysis excludes the use of commonly applied bulk averaging methods due to the fact of their inability to provide for multiscale analysis and also their associated failure to capture the localised information required to study a material’s susceptibility to MC interactions in situ. Atomic force microscopy (AFM) [10] is frequently employed to address these shortcomings, as the technique has the ability to identify localised nanoscale chemically mapping through highly sensitive in situ mechanical force measurements. However, AFM suffers from tip-related limitations and is constrained for MC applications by virtue of its inability to perform quick and user-friendly real-time multiscale analysis. Other techniques that do allow for in situ mapping, such as energy-dispersive X-ray analysis (EDX) and electron backscatter diffraction (EBSD), do not allow for true surface analysis to provide chemical bonding information. For surface engineering this lack of information is a significant limitation, particularly considering that both EDX and EBSD have the benefit of being applied within a scanning electron microscope (SEM), an instrument that has benefitted from the development of highly sensitive commercial in situ mechanical testing stages. Such stages capture data in real time as a sample material is undergoing applied mechanical stress. Combining a surface sensitive chemical imaging technique, housed in an SEM, with the deployment of an in situ mechanical testing stage would be an ideal test environment to reveal mechanochemical effects.

Secondary electron hyperspectral imaging (SEHI) is a multiscale material characterisation technique applied within a scanning electron microscope (SEM). SEHI is based on the collection of secondary electron (SE) emission spectra [11,12,13,14,15] at low primary beam energies, making the technique applicable to the chemical inspection of uncoated polymer surfaces [4,16,17,18,19,20,21,22] as well as metals [23]. SE spectra for some hydrocarbon materials have been found to be influenced by the excitation of intramolecular vibrations [20,21]. SEHI has been applied for the analysis of a range of beam sensitive materials and has been shown to possess the ability to provide high-resolution chemical maps [17,22]. Recently, the SEHI technique was applied to the evaluation of polypropylene surgical mesh and through the data captured provided evidence of the technique’s ability to identify nano–micron scale MC interactions. This study demonstrates that SEHI can provide in situ MC information using poly(glycerol sebacate)-methacrylate (PGS-M) as an example biomaterial of interest [24]. PGS-M is an elastomeric degradable polymer and a functionalised form of the well-studied poly(glycerol sebacate) [25]. This study brings the use of a novel in situ SEM holder together with the application of SEHI to provide, for the first time, enhanced biomaterial characterisation including surface chemical spectroscopy and imaging methods at the multiscale, all of which are considered essential to provide the fundamental analysis needed to evaluate the effects of mechanochemical interactions.

## 2. Materials and Methods

*Synthesis of polyglycerol (sebacate)-methacrylate (PGS-M):* The low molecular weight PGS-M polymer was fabricated following a previously published protocol [24]. In brief, the PGS prepolymer was synthesised by mixing 1:1 (mol/mol) glycerol and sebacic acid, using a hot plate at 120 °C at 300 rpm for 48 h. Nitrogen gas was applied for the first 24 h, and then a vacuum was applied to the system for another 24 h to remove the water from condensation. To methacrylate the PGS prepolymer, 1:4 (*w/v*) dichloromethane (DCM) was used to dissolve the prepolymer. Subsequently, the system was changed to 0 °C in dark condition at 300 rpm, after which 1:1 (mol/mol of PGS hydroxyl groups) of triethylamine (TEA) and 1 mg/g PGS hydroxyl group of 4-methoxyphenol (MeHQ) were added to the system. Methacrylate anhydride (MAA) was used to control the percentage of methacrylation; in this instance, 0.5 mol of MAA was added per mol PGS hydroxyl groups. After 24 h of methacrylation, 30 mM hydrochloric acid was used to wash the PGS-M polymer. The water from reaction was then removed using CaCl2. DCM was removed by rotary evaporation. To synthesis PGS-M, the remaining DCM was removed from PGS-M polymer using a vacuum. Seventy percent PGS-M in DCM was blended with 1:1 (*w/w*) toluene, 10% HypermerTM B246 and 25% diphenyl(2,4,6-trimethylbenzoyl) phosphine oxide/2-hydroxy 2-methylpropiophenone and blended (photoinitiator) at 350 rpm. After 5 min of blending, 4 mL dH_2_O was added dropwise to the emulsion. The emulsion was then photocured for 5 min each side and washed with methanol for 4 days and dH_2_O for 4 days. The PGS-M samples were then exposed to low-pressure argon glow discharge in a Diener Electronic Zepto plasma cleaner at 40 kHz at 100 W for 4 min in a Tyrex gas semipermeable packaging.

*Use of a bespoke SEM holder:* PGS-M samples of 50 (length) × 10 (width) × 5 mm (height) were prepared. The samples were then carefully placed into the holder without any extensive prestress. The PGS-M samples underwent a 140 degree flexion. The three-point SEM holder mitigated sample charging by grounding the samples from contact with aluminium pins. A solder strip was included on the printed circuit board (PCB) to create a current pathway across the copper layers. This allowed the current to dissipate through the aluminium stub inserted into the SEM stage. Adjusting the pin position allowed the user to alter the degree of flexion.

*Conventional low-voltage SEM imaging:* Observation of the surface morphology of the PGS-M was performed using a scanning electron microscope (Helios G4 CX Dual-Beam). The samples were not subject to conductive coatings, in contrast to usual polymer SEM analysis. To reduce surface charging and consequent damage to the sample, a low accelerating voltage of 1 kV with a typical vacuum pressure of 10^−6^ mbar at a working distance of 3 mm was applied. An Everhart Thornley detector (ETD) for low-magnification images and a through-lens detector (TLD) for high-magnification images were selected for the collection of SE images.

*SEHI data collection and processing:* The methodology of the application of SEHI has been published in depth previously [23,24,25,26]. Briefly, SE spectra generation was performed on PGS-M using the Helios FEI Helios G4 CX DualBeam microscope by applying consistent operating conditions of 1 kV and a 50 pA immersion mode. No conductive coating was applied to the samples in contrast to typical SEM imaging. A typical vacuum pressure of ~10−6 mbar, working distance of 3.0 mm, and an accelerating voltage of 1 kV were applied in the immersion mode. The collection of SE spectra of different energy ranges was enabled through the adjustment of the mirror electrode voltage (MV) together with a tube bias setting of 150 V. Stepping the MV in a range of −15 and 15 V (energy range of −0.7 to 12.7 eV) was achieved through the use of an automatic iFast collection recipe [27]. Every image was captured at a frame interval of 0.5 s and an MV step size of 0.5 V, which corresponds to a ~0.2 eV electron energy step size. Image processing was undertaken using Fiji ImageJ software (*ImageJ2, open source*).

## 3. Results and Discussion

**Figure 1:**
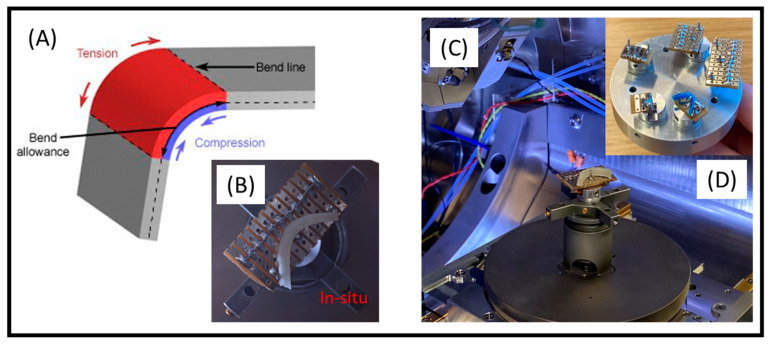
(**A**) Schematic outlining the forces present when implementing a three-point bending test; (**B**) a Nav Cam image taken within a scanning electron microscope (SEM) chamber of a bespoke SEM sample holder subjecting PGS-M to both compression and tension; (**C**) an image showing the bespoke SEM holder attached onto an SEM stage; (**D**) an image showing the range of differing SEM holders with adjustable angles for material flexion.

To showcase SEHI’s capacity to reveal MC altercations, a bespoke SEM holder was manufactured (see Figure 1). The in situ SEM sample holder was prepared with the aim of exposing a specimen to both compression and tension forces (outlined in Figure 1A). The holder produced adopted the principles of a traditional three-point bending test, thereby allowing the material to experience in situ compression as well as tension (Figure 1B). The three-point SEM holder was developed to mitigate sample charging by grounding the sample via contact with aluminium pins. A solder strip was added to the PCB to create a current pathway across the copper layers; this allowed the current to dissipate through the aluminium stub inserted onto the SEM stage (Figure 1C,D). Mitigating sample charging is essential for the analysis of beam sensitive materials such as poor conducting polymers. 

**Figure 2:**
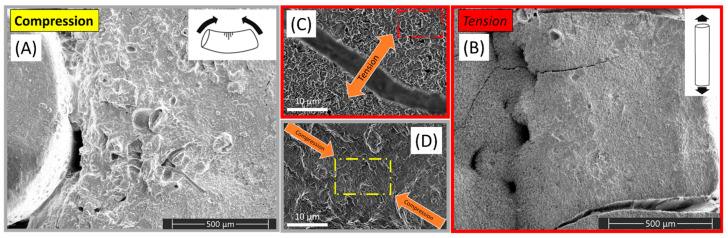
(**A**) A conventional SEM image of PGS-M under compression taken using an Everhart Thornley detector (ETD); (**B**) a conventional SEM image of PGS-M under tension taken using an Everhart Thornley detector (ETD); (**C**) a secondary electron (SE) image of PGS-M under tension taken using a through-lens detector (TLD) at a horizontal field of view of 25 μm; (**D**) a secondary electron (SE) image of PGS-M under compression taken using a through-lens detector (TLD) at a horizontal field of view of 25 μm.

As the SEM images indicate, areas of the PGS-M were subject to either compression or tension stress as predicted in Figure 1. Figure 2A displays a conventional SEM image of the material under compression, showing polymer buckling under the stress provided by flexion occurring around the pin, shown at the right-hand side of the SEM image. In contrast Figure 2B shows a conventional SEM image of the same material taken from the other side of the sample where the force of tension was dominant on the material’s surface. On this surface, longitudinal cracks can clearly be seen occurring as a result of the high-tension force applied. From the conventional SEM images alone, it is noticeable that the surface morphology displayed significant differences in response to the force being applied across the material. The insets in Figure 2 (Figure 2C,D) show that for high-tension and high-compression regions, it was also clear that the images showed differences in nano–micron features. For the high-tension region, micron-scale cracking is visible, whereas the high-compression region shows what appear to be a more sheer stress-related morphology and crushing.

**Figure 3:**
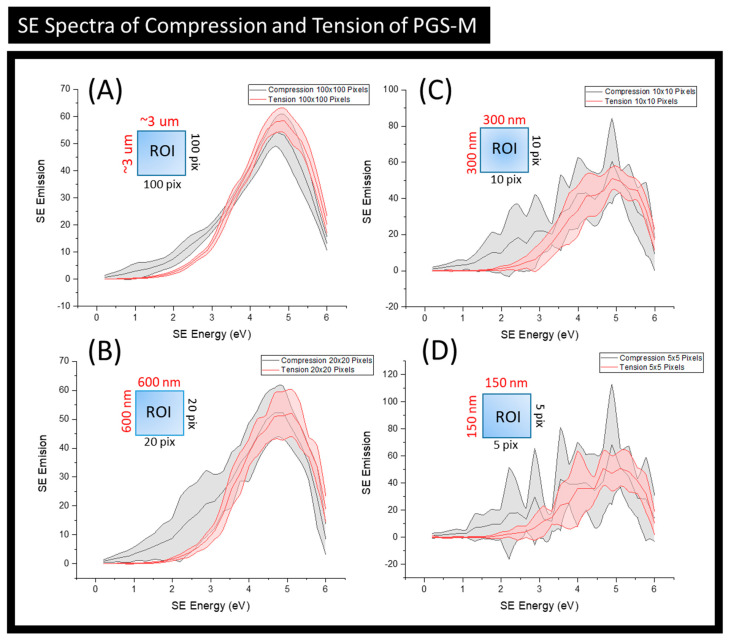
(**A**) Secondary electron spectra (SES) for regions of PGSM under compression (*n* = 6) and tension (*n* = 6). SE spectra collected from an ROI of 3 × 3 μm using the Helios FEI Helios G4 CX DualBeam microscope. (**B**) SES for regions of PGS-M under compression (*n* = 6) and tension (*n* = 6). SE spectra collected from an ROI of 600 × 600 nm using the Helios FEI Helios G4 CX DualBeam microscope. (**C**) SES spectra for regions of PGS-M under compression (*n* = 6) and tension (*n* = 6). SE spectra collected from an ROI of 300 × 300 nm using the Helios FEI Helios G4 CX DualBeam microscope. (**D**) SES spectra for regions of PGS-M under compression (*n* = 6) and tension (*n* = 6). SE spectra collected from an ROI of 150 × 150 nm using the Helios FEI Helios G4 CX DualBeam microscope.

In order to assess any chemical changes taking place while the PGS-M underwent compression and tension, SE spectra were collected and are presented in Figure 3. To highlight the multiscale nature of SEHI, four different sized regions of interest (ROIs) were analysed thus allowing for micron–nanoscale information to be obtained. To facilitate this, SEHI data collection was configured so that each pixel exhibited a 30 nm resolution. The largest ROI (Figure 3A) averaged an SE emission over 3 μm^2^ (100 × 100 pixels) to generate the resulting SE spectra presented. This is in contract to the smallest ROI (Figure 3D), which averaged over 150 nm^2^ (5 × 5 pixels). All of the SE spectra presented showed that PGS-M surfaces under compression differed markedly to that of PGS-M under tension. Of interest was that the variation of difference was masked at larger ROIs (Figure 3A,B) when compared to that of smaller ROIs (Figure 3C,D). This highlights the importance of applying multiscale analysis methodologies due to the potential of data smoothing out important information if the focus of the analysis is based on a single-length scale. As the area of the ROI was reduced, finer SE peak structures became apparent, and of interest was the increased SE emission variation within 4–6 eV. This range has been shown to contain signatures of chemical groups containing oxygen functionalities [4,17,22]. This finding was expected, as it has been shown that mechanical force has the potential to alter chemical bonding [28]. Previous studies observed that the localised bond breaking leads to a strong increase in oxygen-containing end groups through chain scission [29]. Such an effect of polymer oxidation has been found to be stronger at the surface, compared to bulk material, and is explained as being the result of surface bonds being exposed to higher local loads and higher concentrations of oxygen [4]. Identifying such surface chemistry variants is important in the development of biomaterials, as it is well established that an increase in O-containing functionalities is proportional to improved cellular growth [30].

The most notable difference between the compression and tension ROIs, at all length scales observed, was within the SE emission region of 1.4–2.3 eV. This range has previously been identified as having a relationship to molecular order/density [16,21]. The SE spectra indicated that compression of PGS-M increased the molecular density of the material ROI compared to that of the PGS-M under tension. Changes in molecular density and order are well-established material effects of MC. In solid materials, the result of mechanochemical reactions is the local build-up of radicals. Depending on the concentration of free radicals, the molecular order can locally increase at high concentrations, until crosslinking occurs or it decreases at low radical concentrations [31]. This finding is in line with expectations due to the fact that the compression of a material’s ROI’s volume will result in an increase in its density (density = mass/volume); however, this result does shows the capacity of SEHI to observe such molecular differences across PGS-M samples when subjected to different stress conditions. MC reactions require the input of kinetic energy; in all cases, this input is localised across a material. The multiscale localised data presented validates the known effect that kinetic energy causes localised molecular chain extensions. This process yields the free radicals required for polymer chain oxidation and molecular density realignment [29,31].

**Figure 4:**
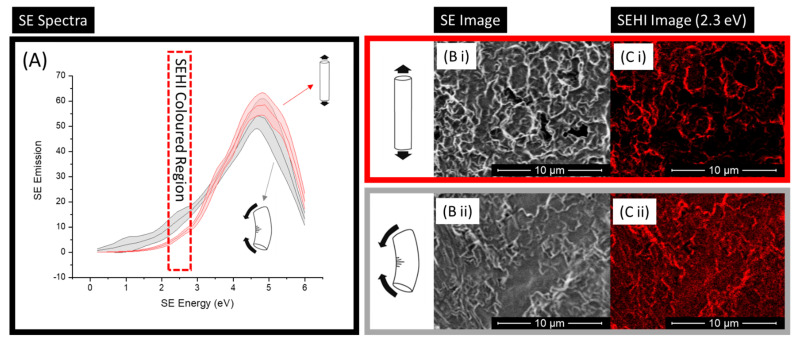
(**A**) Secondary electron spectra (SES) for regions of PGSM under compression (*n* = 6) and tension (*n* = 6) collected using the Helios FEI Helios G4 CX DualBeam microscope. The SE spectra highlights an energy window of 2.4 eV selected for SEHI mapping. (**B**) Secondary electron (SE) images of PGS-M under (**i**) tension and (**ii**) compression, taken using a through-lens detector (TLD) (10 μm scale bar). (**C**) Secondary electron hyperspectral imaging (SEHI) image of PGS-M under (**i**) tension and (**ii**) compression, taken using a through-lens detector (TLD) (10 μm scale bar).

Aside from the generation of SE spectra, SEHI has the ability to map chemical differences within the SE ranges identified. Previous studies have shown that through the application of a non-negative matrix factorisation (nnmf) approach [16,17,22], SEHI can select energy ranges from resulting SE spectra without any user input or bias. This is the process that was applied to match various SE peak locations to the expressing chemical bond/group [4,22]. From such SEHI maps, it is possible visualise the homogenous/heterogeneous nature of polymers [16,21]. In this instance, SEHI mapping was applied only to the region of the SE spectra associated with molecular density (1.4–2.3 eV). Figure 4 shows this process, starting with the SE spectra displayed in Figure 4A, highlighting the region to be coloured, with the resulting colour SEHI map shown in Figure 4C. For ease of comparison, an SE image (Figure 4B) is also included as an example, showing how specific energy regions show identifiable SE emissions. These SEHI maps further the interpretability of obtained SE spectra results, clearly showing that there was a large variation in localised intensities for molecular order SE emissions. It is notable that there was greater intensity for the compression region compared to that of the tension region of PGS-M. As previously highlighted, this was an expected finding. Of interest, however, is that the SEHI images presented (Figure 4C) show that the orientation of force created highly ordered molecular regions following the path of the force applied. For example, within the tension region’s SEHI image, it is noticeable that highly dense polymer chains are more commonly apparent from the top to the bottom of the image (following the stress of tension), whereas the compression image shows high-density chains aligned corner to corner in the SEHI image. For biomaterials, it has been long recognised that a material’s mechanical properties play a key role in the adhesion of cells onto a biomaterial’s surface [32]. An implanted polymer-derived biomaterial will, in almost all deployment scenarios, undergo some form of mechanical stress in situ [33,34,35]. Such a force will certainly not be uniform in load and will result in localised stresses occurring across the material’s bulk and surface [36]. With prestress mechanical testing, it is possible to capture bulk mechanical failures as a result of a preloading stress [37]. However, for localised surface stresses, which alter surface mechanical properties and, in turn, cellular adhesion, Figure 4C shows that SEHI has the capacity to map spatial variations in molecular density at length scales that are directly applicable to biomaterial integration.

## 4. Conclusions

This study highlighted the use of a novel in situ SEM holder combined with the application of SEHI to provide, for the first time, enhanced biomaterial MC characterisation. The results obtained showed the capacity of SEHI to observe chemical and molecular differences across PGS-M samples when they were subjected to different stress conditions. Both the SE spectra and SEHI maps presented provide information on the localised variation of the effect of MC raised by compression and tension stress. Future work should build upon this static-induced stress study by implementing SEHI with a dynamic in situ mechanical stage. Such a capacity to perform dynamic chemical/mechanical characterisation would uniquely inform how the process of deformation under stress influences a sample material’s localised chemistry, not only for polymer-derived biomaterials but also for implementing SEHI to evaluate biomedical metal implants and surface coatings. This test capability would enable researchers to unlock a new understanding of a material’s suitability and, importantly, its limitations within the intended deployment environment. This could be achieved through characterisation analysis being undertaken within an environment that is representative of the dynamics of the material’s application environment. For the field of biomaterials, more effective mechanochemical assessment is expected to facilitate the elimination of unsuitable material candidates prior to extensive, time-consuming and expensive clinical testing, thus helping to promote the development and quality assurance of the next generation of medical implants.

## Data Availability

Data available from https://doi.org/10.15131/shef.data.19723468 accessed on 5 March 2022.
